# Children with cancer: a survey on the experience of Italian primary care pediatricians

**DOI:** 10.1186/s13052-017-0365-9

**Published:** 2017-05-25

**Authors:** Marta Minute, Giorgio Cozzi, Chiara Plotti, Giuseppe Montanari, Paolo Pecile, Giulio Andrea Zanazzo, Alessandro Ventura, Egidio Barbi

**Affiliations:** 1 Institute for Maternal and Child Health IRCCS Burlo Garofolo, via dell’Istria 65/1, Trieste, Italy; 2 University of Trieste, piazzale Europa 1, Trieste, Italy; 3 Azienda per l’Assistenza Sanitaria numero 5 Friuli Occidentale, Via della Vecchia Ceramica 1, 33170 Pordenone, Italy; 4 Azienda per l’Assistenza Sanitaria numero 4 Friuli Centrale, via Pozzuolo 330, 33100 Udine, Italy

**Keywords:** Childhood cancer, Pediatric primary care, Pediatric oncology

## Abstract

**Background:**

Cancer is the second cause of death in children and its diagnosis can be difficult, due to the presence of vague and non-specific symptoms. The primary care pediatrician is often involved in the diagnostic process, but no longer in child care once the treatment started. Care models involving both primary care pediatricians and oncologic referral centre highlighted a higher family satisfaction when they worked together. We conducted a survey on primary care pediatricians involved in childhood cancer in order to describe the actual situation.

**Methods:**

We conducted a retrospective survey enrolling primary care pediatricians from a north-eastern area of Italy. They received a questionnaire that consisted in two parts: the first one aimed to assess the physician’s seniority and experience and the second one pertained to each case of cancer and explored the relationship between the pediatrician, the family and the referral centre, and pediatricians degree of satisfaction and emotional impact.

**Results:**

We obtained data from 79 pediatricians who described 150 cancer cases. In 99 cases the primary care pediatrician had visited the child at the onset of symptoms and had referred him to the hospital. In 89 cases, he understood the severity of the disease. In 53.3% of cases the pediatrician was informed by the referral centre. The relationship between the pediatrician and child’s family improved in 38% of cases and this was related with their participation to the multidisciplinary meetings on child health.

**Conclusions:**

Primary pediatricians’ sharing in the management of their patients with cancer was not satisfactory. Development of specific protocols targeted to an integrated care is needed to increase primary pediatricians’ involvement and families’ satisfactions.

**Electronic supplementary material:**

The online version of this article (doi:10.1186/s13052-017-0365-9) contains supplementary material, which is available to authorized users.

## Background

The annual incidence of cancer in childhood is about 15/100.000 pediatric patients [1].

A primary care physician can expect to meet an average of two children with cancer during his or her career [2]. Despite being quite a rare event, cancer is the second cause of death and the first medical cause of death in children between five and fourteen years of age [3]. The most common type of cancer are blood cancers (49%) and central nervous system tumours (18%) [1].

Children with cancer often present with vague symptoms, that can overlap with common complaints of childhood [4]. For this reason, the diagnosis often relies on a combination of discrete symptoms rather than on a single key sign [5], thus emphasizing the role of the primary care pediatrician in detecting something wrong, often supported by the suggestion of parents [6].

Not rarely, after diagnosis, the primary care paediatrician is no longer involved in the care of the patient, no matter whether he or her contributed to the diagnosis or if the diagnostic process was started elsewhere (emergency department, second opinion) [7].

Ideally, as stated by the American Academy of Pediatrics [8], primary care pediatricians come back to their role when the aggressive care phase ends, being involved both in follow-up clinical assessments of previously treated patients that might face long term consequences of their therapies [9, 10] and in palliative care of those children who end their life at home [11].

The gap between the referral centre and the primary care pediatrician disappoints families and children who feel abandoned. Referral centres are frequently overwhelmed by requests that could be managed by primary care pediatricians who, on the other hand, complain that they are not informed on their patients’ course of the disease [7].

Only a few data are available on primary care pediatricians’ experience with childhood cancer, particularly about their relationship with the families and the referral centre. We conducted a survey on primary care pediatricians. Our aim was to describe the reality of a defined area, according to primary care pediatricians’ experience.

## Methods

Between March and June 2016, we conducted a retrospective survey enrolling primary care pediatricians of Friuli Venezia Giulia, a north-eastern area of Italy, where the total population amounts to 1.228 million and the pediatric (0–14 years of age) to 150.000. In this area, the mean incidence of childhood cancer is almost 14/100.000/year.

Each of the 118 primary care pediatricians working in this area received a non-validated questionnaire (Additional file 1) that consisted in two parts: the first one aimed to assess the physician’s seniority and experience and the second one pertained to each case of cancer and explored the relationship between the pediatrician, the family and the referral centre, and the primary care pediatrician’s degree of satisfaction and emotional impact. The questionnaire was sent by email to the address registered in a regional database and it was anonymous. Even physicians who did not have had patients with malignancies were invited to participate to the first part of the questionnaire. If the doctor had two or more patients with cancer, a file for each patient was provided.

### Statistical analysis

Quantitative variables were summarized using mean value and standard deviation. Qualitative variables were expressed as absolute numbers and percentages. Any differences between categorical data were assessed through the Fisher exact test. The differences between the data expressed as a percentage were evaluated using the tests of proportions. In order to test the correlation between the number of cancer patients and the years of experience of the primary care pediatrician we used the Spearman rank correlation coefficient. The statistical significance criterion was set for a *p*-value <0.05. For data analysis was used the Excel spreadsheet and the version 3.0.3 R software (2014).

## Results

We obtained data from 79/118 (67%) primary care pediatricians who described a total of 150 cases of children affected with childhood cancer.

The pediatricians’ data and work experience are shown in Table 1.

**Table 1:** Primary care pediatricians (*n* = 79)

Age, years (mean, SD)	56.5 +/− 7.8
Sex, M (M %)	26 (33%)
Sex, F (F %)	53 (67%)
Years of activity as primary care pediatrician (mean, SD)	20 +/− 9.3
Number of patients (mean, SD)	994 +/−154
Number of patients with malignancy (mean, SD)	2.6 +/− 2.1
Usefulness of a refreshing course on childhood cancer, number (%)	Yes: 70 (88.6%)
	No: 6 (7.6%)
	No answer: 3 (3.8%)

The correlation between the pediatricians’ years of activity and the number of cancer cases was not linear although it was statistically significant (Spearman correlation coefficient = 0.38, *p* = 0.0005). The estimated number of patients with cancer for each paediatrician was 0.17/year.

**Fig. 1:**
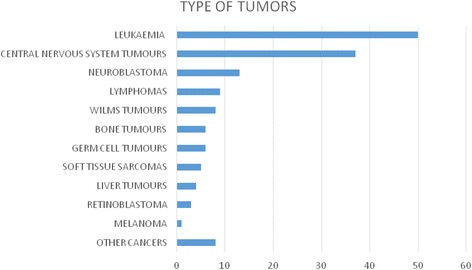
Type of cancer diagnosed in children in our sample

Considering all the reported cancer cases, the mean age at diagnosis was 5.36 +/− 3.8 years. Ninety-four (62.6%) patients were males. The most frequent cancers are summarized in Fig. 1.

We obtained data about the onset of symptoms of 113 patients. In 99 cases (87.6%) the primary care pediatrician had visited the child at the onset of symptoms. In 93 cases (82.3% of the sample, 94% of those who were visited by the pediatrician) the patient was referred to the hospital for further investigation. In 14 cases (12.4%) the child was taken directly to hospital by parents, due to acute onset of symptoms. In 89 cases (78.7% of the sample, 89.9% of those who were visited) the primary care pediatrician understood the severity of the case, while the parents understood the severity in 51 cases (45.1%).

**Fig. 2:**
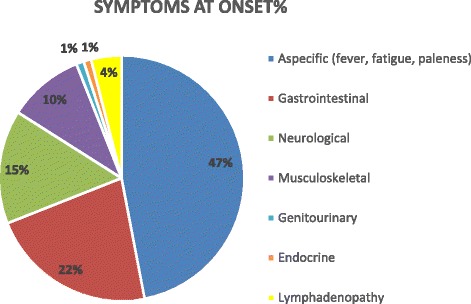
Symptoms at onset

Onset symptoms of the disease are described in Fig. 2. The main onset symptoms are the non-specific ones, such as fever, weight loss, weakness, pallor and pain.

Time between symptoms onset and cancer diagnosis ranged from a minimum of a few hours to a maximum of 1095 days; the median time was 14 days.

The mean time to diagnosis in leukemia was 16.2 days (range 1–150 days, median 7 days) while a mean of 87 days was necessary to diagnose a solid tumour (range 1–1000 days, median 20 days).

Data about the relationship between primary care pediatrician and the families are summarized in Table 2.

**Table 2:** Relationship between primary care pediatrician and families after cancer diagnosis (*n* = 150)

Trend in relationship with family, number (%)	Improved: 49 (32.7%)
	Unchanged: 65 (43.3%)
	Worsened: 14 (9.3%)
	No answer: 22 (14.6%)
Trend in patient accesses, number (%)	Increased: 56 (37.3%)
	Decreased: 75 (50%)
	Unchanged: 19 (12.7%)
Involvement in multidisciplinary meetings, number (%)	Yes: 39 (26.0%)
	No: 88 (58.7%)
	No answer: 23 (15.3%)
Involvement in child home care, number (%)	Yes: 29 (19.4%)
	No: 98 (65.3%)
	No answer: 23 (15.3%)

A correlation between the improvement of the family-pediatrician relationship and the pediatrician’s participation to the multidisciplinary meetings on child health was noted and was statistically significant (*p* = 0.007).

Data about the relationship between primary care pediatrician and oncologic referral centre are summarized in Table 3.

**Table 3:** Relationship between primary care pediatrician and oncologic referral centre (*n* = 150)

Information about the diagnosis, number (percentage)	Oncologic referral center: 80 (53.3%)
	Parents: 58 (38.7%)
	None: 5 (3.4%)
	No answer: 7 (4.6%)
Satisfaction rate (mean, SD) [range 1–10]	6.25 (+/− 2.6)

Primary care pediatricians referred to have received information about the course of patients’ disease by letter (carried by the parents) in 34.2% of cases, by phone in 30.9% of cases and by email in 13.8% of cases. In 21% of cases no information was provided.

The emotional involvement reported by the primary care pediatricians was expressed by means of a numerical rating scale of one to ten, with ten being the highest level of involvement. The mean value proved to be 8.6 +/− 1.4.

Seven primary care pediatricians had been involved in the terminal care of their patients, out of the 20 patients who died at home.

## Discussion

This study showed a strong disparity between the role of the primary care pediatrician in the diagnosis of cancer and the subsequent sharing of the management of the affected children. In this experience, primary care physicians were involved in more than 70% of the diagnostic processes, but only 20% were steadily involved in child care once the diagnosis was set, despite the evidence that an integrated approach improves family experience with the disease [7].

To the best of our knowledge, this was the first paper that investigated the perception of the primary care pediatrician, except for an Italian survey conducted in the same area in 2001, that demonstrated comparable results [12].

We obtained data from 79 pediatricians (out of 118) and 150 cases of childhood cancer has been described, consistently with the literature data, according to which a primary care physician meets an average of two childhood cancer patients [2]. We estimated that each pediatrician met 0.17 children with cancer/year.

Our results were consistent with the literature in describing male sex predominance and leukemia and central nervous system cancers as the most frequent childhood malignant tumours [1]. As reported in the literature, symptoms that lead to diagnosis were vague and unclear also in this series without a consistent delay on diagnosis, that was timely suspected by the involved primary care pediatrician in 72.3% of the cases.

Considering the relationship between the primary care physician and the family, it improved in 38% of cases and deteriorated in 10%, without a linear correlation with the severity and the outcome of the disease. Since there are no data in the literature we cannot compare these results, further studies should address this issue and identify factors improving the quality of the relationship between the primary care physician and the family.

The primary pediatricians involvement in multidisciplinary meetings, established to take global care of the sick children was associated with an improvement of the relationship with the family(*p* = 0.007).

Concerning terminal palliative care, we were aware of only seven primary care pediatricians who had been involved; they reported this experience as being extremely touching and enriching, since it created a strong connection with both the child and the family in a domestic environment. They all underlined the need of a specific training provided by the referral centre.

The relationship between primary care and referral centre appeared to be critical: 21% of primary care pediatricians reported a total absence of communication. The majority declared to be unsatisfied with the relationship with the referral centre, whose efficacy depended more on personal connections between colleagues than on standardized protocols.

These difficulties might depend on different variables, first of all to the fact that the referral centre and the primary care pediatrician are not the only actors in the care of childhood cancer, since peripheral hospitals and local home care are involved, and often families prefer to lean on those structure where they feel more protected than on their physician.

This survey had some limitations in its generalizability. First, we considered only a specific geographic area and we obtained answers from 67% of the pediatricians; it might be possible that those who didn’t answer the survey had had a worse experience. Moreover, our sample was represented by a heterogeneous population of primary care pediatricians and data had been collected altogether, without considering the period in which the patient had been taken care of: considering that some pediatricians had been working for more than thirty years it could have been useful to stratify our sample, as the management of childhood cancer has deeply changed in recent years and some recall bias could have happened when describing the older cases.

Another limitation consisted on the fact that, due to data collection, we hadn’t been able to compare patient/pediatrician’s relationship according to diagnosis and duration of treatment, which might be extremely different in the study group. A subgroup analysis in a larger cohort could be interesting to stratify our results in a more homogenous population.

Our paper shows that the primary physician’s perception of the relationship with the families and the referral centre has significant margins of improvement with the most critical issues being the perceived lack of communication and cooperation with the referral centre. Since a better quality of the relationship between primary care physicians and families has been shown to be associated with an improved quality of care, specific efforts should address this issue. Further studies should investigate the attitudes of referral oncologists and of parents of children with cancer, to identify communication pitfalls and areas of intervention to improve the global care of pediatric cancer patients.

## Conclusion

In conclusion, in this study primary pediatricians feel that the management of their patients with cancer was not satisfactorily shared. Considering that the participation of the primary care physician in multidisciplinary meetings was strongly associated with an improvement of the relationship with the family, we think that development of specific protocols targeted to an integrated care is needed to increase primary pediatricians’ sharing and families’ satisfactions.

## Additional file

Additional file 1: Questionnaire. (DOC 39 kb)
