# Supplementary material for: Children with cancer: a survey on the experience of Italian primary care pediatricians
Source: Ital J Pediatr. 2017 May 25;43:48. doi: 10.1186/s13052-017-0365-9 (PMC5445274; doi:10.1186/s13052-017-0365-9)
Supplement: Additional file 1: — Questionnaire. (DOC 39 kb) [file 13052_2017_365_MOESM1_ESM.doc]

**Additional file 1**

**QUESTIONNAIRE**

**PART 1: PEDIATRICIAN’S CAREER**

**AGE: ___**

**SEX:**

◻F

◻M

**YEARS of activity AS PRIMARY CARE PEDIATRICIAN ___**

**NUMBER OF PATIENTS: ___**

**NUMBER OF PATIENTS WITH CHILDHOOD CANCER ____**

**DO YOU CONSIDER A REFRESH ON CHILDHOOD CANCER USEFUL?**

◻ Yes

◻ No

**PART 2: SINGLE CASE**

**AGE AT DIAGNOSIS: ___**

**TYPE OF CANCER:** ___

**SEX:**

◻ F

◻ M

**DID YOU VISIT THE CHILD FOR THE SYMPTOMS THAT LED TO THE DIAGNOSIS?**

◻ Yes, the symptoms were: ______________________________________________________

◻ No Because: ________________________________________________________________

**DID YOU SEND THE CHILD TO THE HOSPITAL OR WAS HE/SHE BROUGHT DIRECTLY FROM THE FAMILY? ___________________________________________________________________________**

**DID YOU UNDERSTAND THE SEVERITY OF THE CASE?**

◻Yes

◻ No Because: _______________________________________________________________

**DID THE PARENTS UNDERSTAND THE SEVERITY OF THE CASE?**

◻ Yes

◻ No Because: _______________________________________________________________

**TIME BETWEEN THE ONSET OF SYMPTOMS AT DIAGNOSIS: ___**

**WHO INFORMED YOU ABOUT THE DIAGNOSIS?**

◻ Parents

◻ Oncologic referral centre

◻ none

**THE RELATIONSHIP WITH THE CHILD’S FAMILY:**

◻ Improved

◻ Did not change

◻ Worsened

**HOW MUCH ARE YOU STATISFIED WITH THE RELATIONSHIP WITH THE ONCOLOGIC REFERRAL CENTRE (1-10)? ____**

**AFTER THE DIAGNOSIS, THE PATIENT’S ACCESSES TO YOUR OFFICE:**

◻ Increased

 Did not change

◻ Decreased

**DID YOU TAKE PART TO MULTI DISCIPLINARY MEETINGS ON CHILD’S HEALTH?**

◻ Yes

◻ No Because: _______________________________________________________________

**WERE YOU INVOLVED IN HOME ASSISTANCE?**

◻ Yes

◻ No Because: _______________________________________________________________

**WERE YOU CALLED DURING THE TERMINAL PHASES OF THE PATIENT’S LIFE?**

◻ Yes

◻ No Because: _______________________________________________________________

**RATE YOUR EMOTIONAL INVOLVEMENT (1-10):** ____
